# Comparative Analysis of Chloramphenicol-Resistant *Enterococcus faecalis* Isolated from Dairy Companies in Korea

**DOI:** 10.3390/vetsci8080143

**Published:** 2021-07-27

**Authors:** Sung Hyun Bae, Sunghyun Yoon, Koeun Kim, Yeong Bin Kim, Young Ju Lee

**Affiliations:** 1 College of Veterinary Medicine and Zoonoses Research Institute, Kyungpook National University, Daegu 41566, Korea; gtroyro@gmail.com (S.H.B.); goguma0707@gmail.com (S.Y.); kke02062@gmail.com (K.K.); kimybins@gmail.com (Y.B.K.); 2 Division of Microbiology, National Center for Toxicological Research, U.S. Food and Drug Administration, Jefferson, AR 72079, USA; 3 Division of Bacterial Disease, Bureau of Infection Disease Diagnosis Control, Korea Disease Control and Prevention Agency, Osong 28159, Korea

**Keywords:** *Enterococcus faecalis*, chloramphenicol resistance, milk, multidrug resistance

## Abstract

Although chloramphenicol is currently banned from use in livestock, other phenicols, such as florfenicol and thiamphenicol, have been used for the treatment of bacterial infections in domestic cattle in Korea. This study compares the characteristics of chloramphenicol-resistant *Enterococcus faecalis* isolated from the bulk tank milk of four major dairy companies in Korea. Although the distribution of multidrug resistance patterns showed no significant differences between the four companies, 85 chloramphenicol-resistant *Enterococcus faecalis* isolates showed a significantly high number of resistances against five or six antimicrobial classes (37.6%, respectively) (*p* < 0.05). When analyzing the distribution of phenicol resistance genes, 31 (36.5%) isolates only carried the *catA* gene, and two (6.3%) isolates from company A only carried the *cfr* gene. No isolates carried the *catB* or *fexA* genes. Regarding the distribution of other resistance genes, both the *tetL* and *tetM* (45.9%), *ermB* (82.4%), and both *aac(6″)-Ie-aph(2″)-la* and *ant(6′)-Ia* genes (30.6%) showed a high prevalence, and the *optrA* and *poxtA* genes were observed separately, each in only two (2.4%) isolates. Our results confirm that the dissemination of chloramphenicol-resistant *Enterococcus faecalis* and some antimicrobial resistance genes show significant differences between dairy companies. Therefore, our results support that each dairy company should undertake effective surveillance programs to better understand and minimize the emergence of resistance on a multidisciplinary level.

## 1. Introduction

Chloramphenicol, a broad-spectrum antimicrobial in human and veterinary medicine in use since the 1950s [1], inhibits translation in bacteria, by inhibiting peptidyl transferase activity, which is mediated by binding to several proteins in the 50S ribosomal subunit [2]. However, since chloramphenicol has been shown to cause serious bone marrow suppression and fetal aplastic anemia [1,3], its use in humans and animals has been subsequently limited. It is currently banned from use in food-producing animals in many countries and regions, including the European Union [1], China [4], the United States [5], and Korea [6].

Enterococci are known as a genus of gut-equivalent microorganisms commonly found in animals and humans, but considered to be environmental mastitis-causing pathogens that can enter into milk and milk products via unhygienic food production and handling conditions [7,8,9]. Moreover, enterococci have been recognized as notoriously opportunistic pathogens that frequently acquire antimicrobial resistance determinants and potential virulence factors [10,11]. Resistance to chloramphenicol is mainly caused by the production of inactivating chloramphenicol acetyltransferase (CAT) [1], the genes of which are widely disseminated on plasmids and capable of contributing to multidrug resistance (MDR) by conjugative transfer [12,13]. Therefore, the occurrence of chloramphenicol-resistant *Enterococcus faecalis* related MDR from dairy products and farms is often reported [14,15,16,17,18]. This study compares the phenotypic and genotypic characteristics of chloramphenicol-resistant *E. faecalis* isolated from the bulk tank milk of four major dairy companies in Korea to provide an insight into the potential emerging problems of *Enterococcus* spp. in milk.

## 2. Materials and Methods

### 2.1. Bacterial Isolation

A total of 1584 batches of bulk tank milk were aseptically collected from 396 farms affiliated with four dairy companies in Korea in the summer and winter seasons. The isolation and identification of *Enterococcus* spp. was performed following standard microbiological protocols published by the Ministry of Food and Drug Safety (Korea) (2019) [19]. Briefly, a one mL milk sample was cultured in nine mL of buffered peptone water (BPW; BD Biosciences, San Jose, CA, USA). Pre-enriched BPW was then mixed with Enterococcosel broth (BD Biosciences, San Jose, CA, USA) at a 1:10 ratio. After incubation at 37 °C for 18–24 h, each medium was streaked onto Enterococcosel agar (BD Biosciences, San Jose, CA, USA). Confirmation of *E. faecalis* was performed via polymerase chain reaction (PCR), with the primer specifically targeted to the *ddl1* gene, as described previously [20]. As a result, a total of 301 *E. faecalis* isolates were tested in this study.

### 2.2. Antimicrobial Susceptibility Testing

Antimicrobial susceptibility analysis was performed by broth microdilution according to the Clinical and Laboratory Standards Institute guidelines (2018) [21], using the commercially available Sensititre^®^ panel KRVP2F (TREK Diagnostic Systems, West Sussex, UK), in accordance with the manufacturer’s instructions. The antimicrobial classes tested were aminoglycosides (gentamicin, kanamycin, and streptomycin), glycopeptides (vancomycin), ionophores (salinomycin), lipopeptides (daptomycin), macrolides (erythromycin, and tylosin tartrate), oxazolidinones (linezolid), penicillins (ampicillin), phenicols (chloramphenicol and florfenicol), quinolones (ciprofloxacin), and tetracyclines (tetracycline and tigecycline). *E. faecalis* ATCC 29212 was used as a quality control. If isolates from the same farm showed the same antimicrobial susceptibility pattern, only one isolate was randomly selected. MDR was defined as acquired non-susceptibility to at least one agent in three or more antimicrobial classes [22].

### 2.3. Detection of Antimicrobial Resistance and Virulence Genes

DNA extraction was prepared by boiling, as described previously [23]. The presence of genes conferring resistance to phenicol (*cfr*, *catA*, *catB*, and *fexA*), tetracycline (*tetL*, *tetM*, and *tetO*), macrolide (*ermA*, *ermB*, and *mef*), oxazolidinone (*optrA* and *poxtA*), and aminoglycoside (*aac(6′)-Ie-aph(2″)-Ia*, *aph(2″)-Ib*, *aph(2″)-Ic*, *aph(2″)-Id*, *ant(3″)-Ia*, and *ant(6′)-Ia*) was investigated by PCR as described previously [24,25,26,27,28,29,30,31,32]. Genes encoding virulence factors, such as collagen-binding cell wall protein (*ace*), endocarditis antigen (*efaA*), aggregation substance (*asa1*), cytolysin (*cylA*), Enterococcal surface protein (*esp*), gelatinase (*gelE*), and pheromone cAD1 precursor lipoprotein (*cad1*) were also detected using PCR, as described previously [33,34].

### 2.4. Statistical Analysis

The SPSS 25 statistical package (IBM corp., Armonk, NY, USA) was used for performing statistical analyses. Pearson’s chi-squared test and Fisher’s exact test with Bonferroni correction were conducted to analyze the differences between the dairy companies, as previously reported [35]. Differences were considered significant at *p* < 0.05.

## 3. Results

### 3.1. Prevalence of Chloramphenicol-Resistant E. faecalis

Among the 301 *E. faecalis* isolates, 85 (28.2%) chloramphenicol-resistant *E. faecalis* isolates were found among the four dairy companies (Table 1). Although company D showed the highest prevalence of *E. faecalis*, the prevalence of chloramphenicol-resistant *E. faecalis* was significantly higher in isolates from company A (61.5%) than company D (12.9%) (*p* < 0.05).

### 3.2. Distribution of Antimicrobial Resistance Patterns

A total of 85 chloramphenicol-resistant *E. faecalis* isolates showed resistance against two to seven antimicrobial classes (Table 2). Although the distribution of antimicrobial patterns showed no significant differences between the four dairy companies, chloramphenicol-resistant *E. faecalis* isolates demonstrated highly significant resistance against five or six antimicrobial classes (37.6%, respectively) (*p* < 0.05). In addition, all chloramphenicol-resistant *E. faecalis* isolates showed the highest resistance to tetracyclines (96.5%), followed by aminoglycosides (92.9%), macrolides (85.9%), lipopeptides (51.8%), quinolones (47.1%), ionophores (38.8%), and oxazolidinones (9.4%). All isolates were susceptible to vancomycin (glycopeptides) and ampicillin (penicillins).

### 3.3. Distribution of Antimicrobial Resistance Genes

The distribution of resistance genes in 85 chloramphenicol-resistant *E. faecalis* isolates is shown in Table 3. In the distribution of phenicol resistance genes, although 52 (61.1%) isolates expressed no resistance genes, 31 (36.5%) isolates carried only the *catA* gene without a significant difference between dairy companies. Two (6.3%) isolates from company A only carried the *cfr* gene, and no isolates carried both the *catB* and *fexA* genes. In the tetracycline resistance genes, the distribution of the *tetM* gene alone (43.5%), and both *tetL* and *tetM* genes (45.9%) was common but showed a significant difference between dairy companies (*p* < 0.05). In the macrolide resistance genes, the prevalence of the *ermB* gene (82.4%) was the highest, and in the oxazolidinone resistance genes, four (4.7%) isolates only carried the *optrA* or *poxtA* genes. In the distribution of aminoglycoside resistance genes, the *ant(6′)-Ia* gene alone (25.9%) and both the *aac(6″)-Ie-aph(2″)-la* and *ant(6′)-Ia* genes (30.6%) were common, without a significant difference between dairy companies.

### 3.4. Distribution of Virulence Genes

The distribution of virulence genes in 85 chloramphenicol-resistant *E. faecalis* isolates is shown in Table 4. Although this distribution showed no significant difference between dairy companies, the most prevalent gene was *ace* (98.8%), followed by *efaA* (97.6%), *cad1* (97.6%), *gelE* (88.2%), *asa1* (62.4%), *esp* (12.9%), and *cylA* (10.6%).

## 4. Discussion

The prevalence of chloramphenicol-resistant enterococci in dairy products has been reported in many countries, including Switzerland (45.9%), Poland (32.91%), South Africa (13%), and Turkey (10.7%) [9,15,18,36]. In this study, the total prevalence of chloramphenicol-resistant *E. faecalis* isolates (28.2%) was also shown similar to other countries, but there were significant differences in the distribution of chloramphenicol-resistant *E. faecalis* between the four dairy companies studied (12.9%–61.5%). In Korea, although chloramphenicol is no longer used in cattle, other phenicols, such as florfenicol and thiamphenicol, have been continuously used for the treatment of bacterial infections in domestic cattle [19]. In particular, florfenicol is commonly recommended for the treatment of bacterial pneumonia and associated respiratory infections caused by *Haemophilus somnus*, *Mannheimia* (*Pasteurella*) *haemolytica*, and *Pasteurella multocida* in cattle [37]. Florfenicol inhibits protein synthesis by binding to ribosomal subunits of susceptible bacteria and shares antimicrobial binding sites with chloramphenicol [1]. Because antimicrobial usage generally results in antimicrobial resistance [5], the varying usage of florfenicol between the dairy companies studied could be attributed to the differences seen in the distribution of chloramphenicol resistance.

In this study, 83 (97.6%) chloramphenicol-resistant *E. faecalis* isolates demonstrated MDR, especially against five or six classes, which showed a significantly higher prevalence. Chloramphenicol resistance mechanisms in bacteria comprise a reduction in membrane permeability, mutations in 23S rRNA, and proliferation of CATs [1]. However, another mechanism of chloramphenicol inactivation is performed by efflux pumps, which are regulated by translation attenuation [1,38]. Efflux pumps can contribute to the internal environment by removing toxins or antimicrobial agents [10,39]; therefore, the efflux of chloramphenicol could simultaneously confer resistance to this antimicrobial as well as others [10,39].

As previously described, the most frequently encountered mechanism of resistance is enzymatic inactivation by acetylation of chloramphenicol via CATs, encoded by the *catA* and *catB* genes, which are widespread among Gram-positive and Gram-negative bacteria [1,12]. Although the *catB* gene has been found in Gram-negative bacteria, the *catA* gene is commonly found in Gram-positive bacteria [1]. Jamet et al. [16] reported that 16 of 20 (80%) chloramphenicol-resistant *E. faecalis* isolates from French cheese carried the *catA* gene. Hummel et al. [14] also reported that all chloramphenicol-resistant *E. faecalis* isolates from milk, whey, and cheese in their study carried the CAT gene. However, only 36.5% of chloramphenicol-resistant *E. faecalis* carried the *catA* gene in this study, indicating that other MDR-related mechanisms, such as efflux pumps, may be involved in chloramphenicol resistance to *E. faecalis* in Korea.

Moreover, chloramphenicol resistance can result from changing the binding site of chloramphenicol in cells through a mutation in 23S rRNA [1], facilitated by the phenicol resistance gene *cfr* [40]. The *cfr* gene is also known as the MDR gene [40]; therefore, several researchers have highlighted increasing concerns with regard to this gene in public health [31,40,41,42]. In addition, the *cfr* gene is thought to be linked to the spread of linezolid resistance [41]. Although Elghaieb et al. [43] and Ahmed et al. [44] reported no *cfr* gene in *E. faecalis* isolates taken from dairy milk, two *cfr*-positive, chloramphenicol-resistant *E. faecalis* isolates were detected in this study. However, these two isolates showed no resistance to linezolid.

Although the distribution of the *catA* gene showed no significant differences between dairy companies, some resistance genes showed a significant difference. The distribution of the *tetM* gene alone and both *tetL* and *tetM* genes, which are related to tetracycline resistance, is common, but showed significant differences between the four companies. Miller et al. [41] reported that the *tetL* gene encodes efflux pumps, which are plasmid-borne determinants. Mobile resistance genes carried via plasmids can be easily transferred by conjugation, which allows the sharing of genetic information such as antimicrobial and virulence genes [45]. In this study, isolates from company B showed the highest prevalence of both *tetL* and *tetM* genes (75.0%), and as a result dissemination of tetracycline-resistant pathogens and MDR, including chloramphenicol may be expected to increase further in company B.

In the distribution of macrolide resistance genes, prevalence of the *ermB* gene (82.4%) was the highest, but also showed significant differences between dairy companies (*p* < 0.05)—isolates from company A and C showed 90.6% and 95.2% *ermB* gene prevalence, respectively, but only 56.3% of isolates from company D carried the *ermB* gene. Moreover, resistance against macrolides was also shown to be higher in isolates from company A (93.8%) and C (95.2%) than company D (62.5%). The mechanisms of macrolide resistance in enterococci are described as two types: (i) inducible resistance associated with methylation of 23S rRNA by a methylation enzyme, and (ii) resistance associated with an efflux pump [41,46]. Although the prevalence of erythromycin-resistant *E. faecalis* from dairy products was reported differently between countries including Korea (85.4%), France (70.9%), Switzerland (60.1%), and Poland (18.5%), most erythromycin-resistant *E. faecalis* isolates were positive for the *ermB* gene [15,16,47,48]. The *ermB* gene, which is considered to be the most widespread macrolide resistance gene among enterococci in livestock and food, induces target modification via the action of methylase, and either conjugative plasmids such as pAMβ1, pRE25, and pUW1965, or transposons, such as Tn*917*, Tn*1545*, Tn*5384*, and Tn*5385*, which carry the *ermB* gene [14,15]. This study suggests that there is a need to further investigate the possibility of mobile resistance gene transfer by sequencing plasmids and detecting genes related to transposon families [49,50].

In this study, eight (9.4%) chloramphenicol-resistant *E. faecalis* isolates from three dairy companies showed co-resistance to linezolid. Surprisingly, four (4.7%) isolates also showed co-resistance to florfenicol and harbored the linezolid resistance genes *optrA* or *poxtA*. Linezolid is considered to be a last resort treatment for infections caused by MDR gram-positive pathogens, including vancomycin-resistant *Enterococcus* spp., methicillin-resistant *Staphylococcus* spp., and *Streptococcus pneumoniae* [51]. Recently, the ABC-F transporter gene, *poxtA*, has been the focus of increasing resistance to phenicols and oxazolidinones [32,52,53]. In addition, a novel gene, *optrA*, confers resistance against linezolid, tedizolid, and phenicols by encoding an ATP-binding cassette transporter [30]. So far, linezolid resistance genes in *E. faecalis* from milk and dairy products have not been reported in Korea. Therefore, as previously reported, there is a need for continuous surveillance to monitor the emergence of linezolid-resistant *E. faecalis* in dairy products, as a matter of public health [43].

In this study, among the four dairy companies studied, most chloramphenicol-resistant *E. faecalis* isolates carried virulence genes including *ace*, *asa1*, *cad1*, *efaA*, and *gelE*, although no significant difference was demonstrated between the companies. The presence of virulence genes may contribute to the severity of pathogenesis and accelerate the transfer of antimicrobial resistance genes, which is of public health concern [18].

## 5. Conclusions

In a comparative analysis of *E. faecalis* isolated from four major dairy companies in Korea, the dissemination of chloramphenicol-resistant *E. faecalis* and some antimicrobial resistance genes showed a significant difference between companies, although the prevalence of MDR showed no significant difference. Therefore, our results support that each dairy company should undertake effective surveillance programs to better understand and minimize the emergence of resistance on a multidisciplinary level.

## Figures and Tables

**Table 1:** Distribution of chloramphenicol-resistant *Enterococcus faecalis* from bulk tank milk in dairy companies.

Dairy Company	No. of Farms ^1^	No. of Isolates	
		*Enterococcus* *faecalis*	Chloramphenicol Resistance (%)
A	106	52	32 (61.5) _a_
B	47	39	16 (41.0) _a,b_
C	120	86	21 (24.4) _b,c_
D	123	124	16 (12.9) _c_
Total	396	301	85 (28.2)

^1^ Bulk tank milk samples were collected each time in the summer and winter seasons by farms. Values in the same column with different subscripts (_a__–c_) differ (*p* < 0.05) for the isolated ratio between the dairy companies.

**Table 2:** Antimicrobial resistance patterns of 85 chloramphenicol-resistant *Enterococcus faecalis* from bulk tank milk in dairy companies.

No. of Antimicrobial Resistance	Resistance Pattern ^1^	No. (%) of Isolates				
		A	B	C	D	Total
		*n* = 32	*n* = 16	*n* = 21	*n* = 16	*n* = 85
2	P-T	1 (3.1)	0 (0.0)	0 (0.0)	0 (0.0)	1 (1.2)
	M-P	0 (0.0)	1 (6.3)	0 (0.0)	0 (0.0)	1 (1.2)
	Total	1 (3.1)	1 (6.3)	0 (0.0)	0 (0.0)	2 (2.4) _c_
4	A-M-P-T	3 (9.4)	0 (0.0)	2 (9.5)	1 (6.3)	6 (7.1)
	M-P-Q-T	2 (6.3)	0 (0.0)	0 (0.0)	0 (0.0)	2 (2.4)
	A-P-Q-T	1 (3.1)	1 (6.3)	0 (0.0)	1 (6.3)	3 (3.5)
	A-O-P-T	0 (0.0)	1 (6.3)	0 (0.0)	0 (0.0)	1 (1.2)
	A-L-P-T	0 (0.0)	1 (6.3)	0 (0.0)	0 (0.0)	1 (1.2)
	I-L-P-T	0 (0.0)	0 (0.0)	1 (4.8)	0 (0.0)	1 (1.2)
	O-P-Q-T	0 (0.0)	0 (0.0)	0 (0.0)	1 (6.3)	1 (1.2)
	Total	6 (18.8)	3 (18.8)	3 (14.3)	3 (18.8)	15 (17.6) _b_
5	A-M-P-Q-T	9 (28.1)	2 (12.5)	4 (19.0)	2 (12.5)	17 (20.0)
	A-I-M-P-T	3 (9.4)	0 (0.0)	2 (9.5)	0 (0.0)	5 (5.95)
	A-L-M-P-T	1 (3.1)	2 (12.5)	0 (0.0)	1 (6.3)	4 (4.7)
	A-I-L-M-P	0 (0.0)	2 (12.5)	0 (0.0)	0 (0.0)	2 (2.4)
	A-O-P-Q-T	0 (0.0)	0 (0.0)	0 (0.0)	3 (18.8)	3 (3.5)
	A-L-P-Q-T	0 (0.0)	0 (0.0)	0 (0.0)	1 (6.3)	1 (1.2)
	Total	13 (40.6)	6 (37.5)	6 (28.6)	7 (43.8)	32 (37.6) _a_
6	A-I-L-M-P-T	7 (21.9)	4 (25.0)	7 (33.3)	4 (25.0)	22 (25.9)
	A-L-M-P-Q-T	4 (12.5)	1 (6.3)	3 (14.3)	1 (6.3)	9 (10.6)
	A-M-O-P-Q-T	0 (0.0)	0 (0.0)	0 (0.0)	1 (6.3)	1 (1.2)
	Total	11 (34.4)	5 (31.3)	10 (47.6)	6 (31.3)	32 (37.6) _a_
7	A-I-L-M-P-Q-T	1 (3.1)	0 (0.0)	1 (4.8)	0 (0.0)	2 (2.4)
	A-I-L-M-O-P-T	0 (0.0)	1 (6.3)	0 (0.0)	0 (0.0)	1 (1.2)
	A-L-M-O-P-Q-T	0 (0.0)	0 (0.0)	1 (4.8)	0 (0.0)	1 (1.2)
	Total	1 (3.1)	1 (6.3)	2 (9.5)	0 (0.0)	4 (4.7) _b,c_

^1^ A = aminoglycosides, I = ionophores, L = lipopeptides, M = macrolides, O = oxazolidinones, P = phenicols, Q = quinolones, T = tetracyclines. Values in the same column with different subscripts (_a__–c_) differ (*p* < 0.05) for the isolated ratios between the antimicrobial resistance classes.

**Table 3:** Antimicrobial resistance genes in 85 chloramphenicol-resistant *Enterococcus faecalis* from bulk tank milk in dairy companies.

Antimicrobial Resistance Gene	No. (%) of Isolates				
	A	B	C	D	Total
	*n* = 32	*n* = 16	*n* = 21	*n* = 16	*n* = 85
Phenicol					
*cfr*	2 (6.3)	0 (0.0)	0 (0.0)	0 (0.0)	2 (2.4)
*catA*	14 (43.7)	4 (25.0)	8 (38.1)	5 (31.3)	31 (36.5)
*catB*	0 (0.0)	0 (0.0)	0 (0.0)	0 (0.0)	0 (0.0)
*fexA*	0 (0.0)	0 (0.0)	0 (0.0)	0 (0.0)	0 (0.0)
None	16 (50.0)	12 (75.0)	13 (61.9)	11 (68.7)	52 (61.1)
Tetracycline					
*tetL*	0 (0.0)	1 (6.3)	2 (9.5)	1 (6.3)	4 (4.7)
*tetM*	19 (59.3) _a_	0 (0.0) _b_	12 (57.1) _a_	6 (37.5) _a_	37 (43.5)
*tetO*	0 (0.0)	0 (0.0)	0 (0.0)	0 (0.0)	0 (0.0)
*tetL + tetM*	11 (34.4) _b_	12 (75.0) _a_	7 (33.3) _a,b_	9 (56.2) _a,b_	39 (45.9)
None	2 (6.3)	3 (18.7)	0 (0.0)	0 (0.0)	5 (5.9)
Macrolide					
*ermA*	0 (0.0)	0 (0.0)	0 (0.0)	0 (0.0)	0 (0.0)
*ermB*	29 (90.6) _a_	12 (75.0) _a,b_	20 (95.2) _a_	9 (56.3) _b_	70 (82.4)
*mef*	0 (0.0)	0 (0.0)	0 (0.0)	0 (0.0)	0 (0.0)
*ermA + ermB*	0 (0.0)	1 (6.3)	0 (0.0)	0 (0.0)	1 (1.2)
None	3 (9.4) _b_	3 (18.7) _a,b_	1 (4.8) _b_	7 (43.7) _a_	14 (16.4)
Oxazolidinone					
*optrA*	0 (0.0)	1 (6.3)	0 (0.0)	1 (6.3)	2 (2.4)
*poxtA*	0 (0.0)	0 (0.0)	1 (4.8)	1 (6.3)	2 (2.4)
None	32 (100.0)	15 (93.7)	20 (95.2)	14 (87.4)	81 (95.2)
Aminoglycoside					
*aac(6″)-Ie-aph(2″)-la*	2 (6.25)	0 (0.0)	0 (0.0)	0 (0.0)	2 (2.4)
*aph(2″)-Ib*	0 (0.0)	0 (0.0)	0 (0.0)	0 (0.0)	0 (0.0)
*aph(2″)-Ic*	0 (0.0)	0 (0.0)	0 (0.0)	0 (0.0)	0 (0.0)
*aph(2″)-Id*	0 (0.0)	0 (0.0)	0 (0.0)	1 (6.3)	1 (1.2)
*ant(3″)-Ia*	0 (0.0)	0 (0.0)	0 (0.0)	0 (0.0)	0 (0.0)
*ant(6* *′* *)-Ia*	5 (15.6)	6 (37.5)	5 (23.8)	6 (37.5)	22 (25.9)
*aac(6″)-Ie-aph(2″)-la + ant(6* *′* *)-Ia*	10 (31.3)	5 (31.3)	9 (42.9)	2 (12.5)	26 (30.6)
*aph(2″)-Ic + ant(6* *′* *)-Ia*	1 (3.1)	0 (0.0)	1 (4.8)	0 (0.0)	2 (2.4)
*aph(2″)-Id + ant(6* *′* *)-Ia*	1 (3.1)	3 (18.8)	2 (9.5)	3 (18.8)	9 (10.6)
*aph(2″)-Ic + aph(2″)-Id + ant(6* *′* *)-Ia*	0 (0.0)	0 (0.0)	1 (4.8)	0 (0.0)	1 (1.2)
None	13 (40.6)	2 (12.4)	3 (14.2)	4 (25.0)	22 (25.8)

_a,b_ Values in the same row with different subscripts differ (*p* < 0.05) for the isolated ratios between the dairy companies.

**Table 4:** Virulence genes in 85 chloramphenicol-resistant *Enterococcus faecalis* from bulk tank milk in dairy companies.

Gene	No. (%) of Isolates					*p*
	A	B	C	D	Total	
	*n* = 32	*n* = 16	*n* = 21	*n* = 16	*n* = 85	
*ace*	32 (100.0)	16 (100.0)	20 (95.2)	16 (100.0)	84 (98.8)	0.624
*efaA*	32 (100.0)	15 (93.8)	20 (95.2)	16 (100.0)	83 (97.6)	0.386
*asa1*	21 (65.6)	12 (75.0)	13 (61.9)	7 (43.8)	53 (62.4)	0.308
*cylA*	4 (12.5)	2 (12.5)	3 (14.3)	0 (0.0)	9 (10.6)	0.493
*esp*	3 (9.4)	2 (12.5)	2 (9.5)	4 (25.0)	11 (12.9)	0.513
*gelE*	29 (90.6)	14 (87.5)	16 (76.2)	16 (100.0)	75 (88.2)	0.173
*cad1*	32 (100.0)	16 (100.0)	20 (95.2)	15 (93.8)	83 (97.6)	0.386

## Data Availability

In regard to the data presented in this study, we included it in the article.
